# Comprehensive Analysis of Next-Generation Sequencing and ARMS-PCR for Detecting *EGFR* S768 Mutations in Chinese Non-Small Cell Lung Cancer Patients

**DOI:** 10.3390/diagnostics16213525

**Published:** 2026-10-27

**Authors:** Binyue Kou, Shuo Chen, Qin Zheng, Danju Luo, Jun Fan, Bo Huang, Xiu Nie

**Affiliations:** Department of Pathology, Union Hospital, Tongji Medical College, Huazhong University of Science and Technology, Wuhan 430022, China

**Keywords:** non-small cell lung cancer (NSCLC), *EGFR*, S768, ARMS-PCR, NGS

## Abstract

**Background:** Accurate detection of mutations at the *EGFR* S768 site is crucial for selecting appropriate treatment regimens for patients with non-small cell lung cancer (NSCLC). This study compared Next-Generation Sequencing (NGS) and amplification refractory mutation system polymerase chain reaction (ARMS-PCR) for detecting *EGFR* S768 mutations in Chinese NSCLC patients. **Methods:** This study retrospectively analyzed 20,731 molecular pathology tests (8345 by NGS and 12,386 by ARMS-PCR). **Results:** The detection rate of *EGFR* S768 variants was significantly lower by ARMS-PCR than by NGS (0.40% vs. 1.15%, *p* < 0.001). The variants detected by ARMS-PCR were predominantly S768I, whereas NGS identified a broader spectrum, including S768I, S768_D770dup, S768_V769delinsIL, and S768_V769insLDS. Furthermore, the *EGFR* S768I mutation frequently co-occurred with other *EGFR* mutations, while insertion mutations at or near the *EGFR* S768 site rarely coexisted with other *EGFR* mutations (95.08% vs. 3.03%, *p* < 0.001). Notably, three cases with S768_V769delinsIL or V769_D770insSVV detected by NGS were identified as S768I by ARMS-PCR, which potentially affected the selection of patients’ treatment plans. The sequence variations such as c.2307_2308insAGCGTGGTG, c.2303_2305delinsTCT, and c.2303_2305delinsTTT may be the reasons for the inconsistency between ARMS-PCR and NGS, due to possible non-specific primer binding. However, this remains a hypothesis based on sequence comparison and requires further experimental validation. **Conclusions:** Complex mutations or insertions around *EGFR* S768 site may cause discordant results between ARMS-PCR and NGS. NGS was recommended to confirm the *EGFR* S768 variants when the patient experiences rapid disease progression during treatment with *EGFR* S768I-directed targeted therapy.

## 1. Introduction

Despite ongoing advances in oncology, lung cancer remains the leading cause of global cancer burden. According to the global cancer statistics for 2022, it accounted for approximately 2.5 million new cases (12.4% of all cancer diagnoses) and 1.8 million deaths (18.7% of total cancer mortality) [1]. The situation in China is even more severe, with 1,060,600 new cases (22.0% of all new malignant tumor cases) and 733,300 deaths (28.5% of all malignant tumor deaths) reported nationwide [2].

Approximately 85% of lung cancer cases are non-small cell lung cancer (NSCLC), with the Epidermal Growth Factor Receptor (*EGFR*) being a key driver gene. Among Chinese patients with advanced lung cancer, the *EGFR* mutation rate is as high as 55.9% [2]. *EGFR* mutations in exons 18–21, most commonly exon 19 deletions (ex19_del) (40–45%) and L858R (35–40%), predict sensitivity to oral EGFR tyrosine kinase inhibitor (TKI) therapy [3]. Exceptionally, different mutation sites and types in *EGFR* exon 20 exhibit varying drug responses, making this a heterogeneous subgroup. For instance, the missense mutation S768I and adjacent insertion mutations at the S768 site in exon 20 affect EGFR function through entirely distinct mechanisms. *EGFR* S768I is an αC-β4 loop mutation that induces a conformational change in the αC-helix and is classified under the P-loop and αC-helix compressing (PACC) subclass [4,5,6,7]. The S768I mutation enhances the stability of the αC-helix through an allosteric regulatory mechanism, thereby promoting EGFR activation [8]. In contrast, insertion mutations at the *EGFR* S768 site (a type of exon 20 insertions, ex20ins), most commonly S768_D770dup, typically occur in the loop region following the αC-helix, forming a “wedge” at the C-helix terminus. This effectively locks the helix in its inward active conformation and creates steric hindrance that impedes binding of numerous TKIs [9,10]. Thus, S768I and adjacent ex20ins alter EGFR structure through distinct mechanisms, leading to different treatment responses. S768I is sensitive to second-generation TKIs, such as afatinib. In contrast, the majority of *EGFR* ex20ins mutations exhibit primary resistance to first- and second-generation TKIs. Currently, for NSCLC patients with *EGFR* ex20ins, amivantamab combined with chemotherapy (carboplatin/pemetrexed) is recommended as first-line treatment by both the Food and Drug Administration (FDA) and the National Medical Products Administration (NMPA) [4,11]; meanwhile, sunvozertinib has also shown promising efficacy in relevant clinical trials [12] and has already been approved by NMPA and FDA by July 2025. Therefore, precise identification of S768 variants is clinically imperative for guiding targeted therapy.

PCR and NGS are two main methods in detecting variants at the *EGFR* S768 site for NSCLC patients. Compared to NGS, PCR offers the advantages of shorter detection cycles and lower costs; however, its detection capability for *EGFR* exon 20 mutations is limited. As reported in several references, missed detections are common with PCR when detecting *EGFR* ex20ins mutations, reflecting its significantly lower detection rate compared to NGS for these specific variants [13,14,15]. Furthermore, the risk of false positives with PCR for S768I deserves greater clinical attention. One study reported that NGS confirmed five of six qPCR-detected cases of *EGFR* S768I variants as false positives, attributed to limitations of the Idylla™ system in primer design and threshold settings or other probable reasons [16]. Another report also described specific false positives in *EGFR* S768I detection using the Idylla™ platform, associated with inherent technical limitations and suboptimal sample DNA quality [17]. These findings suggest that PCR not only carries a risk of missed detections but also has deficits in specificity when detecting *EGFR* S768I. However, the performance of amplification refractory mutation system polymerase chain reaction (ARMS-PCR) system, which is widely used for Chinese NSCLC patients, has not been fully investigated on *EGFR* S768 mutations.

To evaluate the real-world performance of ARMS-PCR for detecting the *EGFR* S768 site in NSCLC, we retrospectively analyzed *EGFR* S768 variants in 12,386 samples tested by ARMS-PCR and 8345 samples tested by NGS. We found some cases with discordant results between ARMS-PCR and NGS platforms. We further analyzed the underlying causes of these discrepancies and assessed the potential implications for clinical decision-making.

## 2. Materials and Methods

### 2.1. Patients and Samples

A retrospective analysis was conducted on 20,731 NSCLC specimens that underwent molecular pathological testing at the Department of Pathology, Union Hospital, Tongji Medical College, Huazhong University of Science and Technology between August 2011 and October 2025. Some patients contributed multiple specimens due to multi-lesions in different sites of lung or repeat sampling and testing before and after treatment. Of these, 12,386 were tested by ARMS-PCR and 8345 by NGS. All the specimens tested here were formalin-fixed paraffin-embedded (FFPE) tissue from surgery, needle biopsy, or pleural effusion sediment cells with ≥10% tumor cells in ratio and ≥100 tumor cells in quantity per slide. Only FFPE tissue with storage less than 3 years was tested to avoid the effect of DNA degradation. All samples included in the final analysis met the minimum quality control criteria required for clinical molecular diagnosis. Samples that failed quality control were excluded. We also reviewed the pathology diagnosis and clinical records of patients with discordant results of *EGFR* S768 variants between ARMS-PCR and NGS to collect clinicopathological features, including age, gender, pathological types, clinical stage, treatment regimens, and follow-up details. Staging was based on the AJCC 8th edition clinical TNM (cTNM) staging system for lung cancer. The study was reviewed and approved by the Ethics Committee of Union Hospital, Tongji Medical College, Huazhong University of Science and Technology (Ethical Approval Number: 2022-0782-01). Written informed consent for participation in this study was obtained from the patients or their adult family members.

### 2.2. ARMS-PCR

Tissue samples were embedded in paraffin and sectioned at 5 μm thickness. The sections were stained with hematoxylin and eosin (H&E) to assess tumor cell content, with ≥10% tumor cells in ratio and ≥100 tumor cells in quantity per slide considered acceptable for quality control. DNA was then extracted using a commercial FFPE DNA extraction kit -Spin Column (Amoy Diagnostics Co., Ltd., Xiamen, China, Version B1.0−4.0). DNA concentration was measured by the Nanodrop UV spectrophotometer or Qubit fluorometer (Thermo Fisher Scientific Inc., Wilmington, DE, USA). DNA was diluted with purified water to the recommended loading concentration specified by the commercial kit (Amoy Diagnostics Co., Ltd., Xiamen, China). The ARMS-PCR assay was performed using the following three kits: AmoyDx^®^ EGFR 29 Mutations Detection Kit (Version P1.0−P8.0), AmoyDx^®^ Pan Lung Cancer PCR Panel of 10 genes Kit (Version P1.0−P6.0), and AmoyDx^®^ Pan Lung Cancer PCR Panel of 11 genes Kit (Version P1.0). All the three kits covered *EGFR* S768I mutation with the following primers: GAAGCCTACGTGATGGCCTT, ACACGTGGGGGTTGTCCACGA, and ACACGTGGGGGTTGTCCACTA, respectively. The version of ARMS-PCR kits has been updated several times from 2011 to 2025, but the primers for *EGFR* S768I have been widely validated since the first version and has not changed in the following versions. The diluted DNA (10 ng/reaction) was then thoroughly mixed with Taq polymerase, and 5 μL of this mixture was pipetted into PCR reaction strips containing the reagents. PCR amplification was performed on a Mx3000P real-time fluorescent quantitative PCR instrument (Agilent Technologies Inc., Santa Clara, CA, USA) following the kit’s amplification protocol. Results were analyzed based on PCR amplification curves, provided that all controls (negative, positive, internal reagent, external reagent) met the quality control requirements. The results were then interpreted according to the specific instructions provided with the kit. This method can detect mutations with frequency as low as 1% (Limit of Detection, LOD) with the analytical sensitivity of 100% and the analytical specificity of 100% according to the manufacturer’s instructions.

### 2.3. NGS

Tumor genomic DNA was extracted from formalin-fixed paraffin-embedded (FFPE) tissue using the BR FFPE DNA KIT (Burning Rock Biotech, Cat. No: BR-ET00102-3, Guangzhou, China). DNA concentration was measured with a Qubit 4.0 fluorometer (Thermo Fisher Scientific Inc., Wilmington, DE, USA). For library preparation, 200 ng tissue DNA was sheared using a Covaris LE220-plus Focused-ultrasonicator (Covaris Inc., Woburn, MA, USA) to achieve fragment sizes of 200–300 bp. The sheared DNA underwent end repair, phosphorylation, and adapter ligation, followed by purification using a Library Preparation Kit (Burning Rock Biotech, HS, DX005-96, Guangzhou, China). Target enrichment was performed by hybridization with capture probes, hybrid selection with streptavidin magnetic beads, and PCR amplification. Library quality was evaluated using the Qubit 4.0 fluorometer with the Equalbit 1x dsDNA HS Assay Kit (Vazyme, Cat. No: EQ121-02, Nanjing, China), and fragment size was assessed by LabChip^®^ GX Touch (PerkinElmer, Shelton, CT, USA) with the Dual Protocol DNA High Sensitivity Reagent Kit (PerkinElmer, Cat. No: CLS760672, Shelton, CT, USA). Sequencing was performed on NextSeq 500 Dx (Illumina, San Diego, CA, USA) with paired-end reads, targeting an average depth of 500×. The library preparation kits used covered 68, 168, or 520 cancer-related genes, including *EGFR* exons 19–21 (Burning Rock Biotech, Guangzhou, China, V1.0−4.3). For sequencing data analysis, Bioinformatic Workflow and Tools Box HBRAS V1.0−V4.2.4.H1 (Burning Rock Biotech, Guangzhou, China) was employed. Briefly, the data were first aligned to the reference human genome (hg19) using the Burrows-Wheeler Aligner v.0.7.10. Then, local alignment optimization, duplication marking, and variant calling were performed using the Genome Analysis Tool Kit v.3.2 especially GATK Mutect2. Variants underwent filtering via the VarScan fpfilter pipeline v.2.4.0, and loci with depth < 100 were filtered out. For single nucleotide variations, a minimum of ten supporting reads was required, while insertion-deletion variations required at least eight supporting reads. Variant substituting cause mutation, while affecting the length of sequence were insertion/deletion. Variants with population frequency > 0.1% in the ExAC, 1000 Genomes, or gnomAD databases were classified as single nucleotide polymorphisms and removed from further analysis. The remaining variants were annotated with ANNOVAR (https://annovar.openbioinformatics.org/en/latest/, 1 February 2016 release, accessed on 1 September 2026) and SnpEff v.3.6. This NGS method can detect mutations with frequency as low as 1% (Limit of Detection, LOD). The version of NGS panel and bioinformatics pipelines has been updated several times (V1.0−V4.3), but all the capture probes and pipelines for calling mutations of *EGFR* exons 18, 19, 20, 21 have not been changed over time because they are hotspot regions and have been widely confirmed in first version.

### 2.4. Data Analysis

R (version 4.5.0) was used for data analysis. Continuous variables were presented as mean ± standard deviation (SD). Age was compared between groups using Student’s *t*-test or Mann–Whitney U test if non-normally distributed. Given the large sample size, *p* values were highly sensitive to small differences. So standardized mean differences (SMD) were also calculated, with SMD < 0.1 considered to indicate adequate balance. Chi-square test was employed for rate comparison. And Fisher’s exact test was used when the expected number in a subgroup was less than 5. *p* < 0.05 was considered as statistically significant. To quantify the magnitude of differences, risk ratios (RR) and risk differences (RD) with 95% confidence intervals (95% CIs) were calculated for rates.

The primary analysis was performed at the specimen level. All submitted specimens were retained, including those from multi-lesions in different sites of lung or repeat sampling and testing before and after treatment, because these samples capture tumor heterogeneity and acquired resistance mutations, which are clinically and biologically meaningful. For the *EGFR* S768I subgroup, the primary analysis was performed at the specimen level. To address the potential non-independence from repeated specimens, a patient-level detection rate analysis was additionally performed, including only the first specimen showing the *EGFR* S768I variant per patient. Sex-stratified analyses were also conducted within this subgroup.

## 3. Results

### 3.1. Baseline Characteristics of Patients and Samples

A total of 20,731 specimens were tested, including 12,386 specimens detected by ARMS-PCR and 8345 by NGS. The two groups were well balanced in age (mean 60.30 vs. 60.93, SMD = 0.060) and sex (female 43.23% vs. 47.67%, SMD = 0.089). Both standardized mean differences (SMD) values were below 0.1, indicating adequate balance between the two groups (Table 1).

### 3.2. Comparison of ARMS-PCR and NGS for Detecting EGFR S768 Variants

The rate of ARMS-PCR detected *EGFR* S768 variants was 0.40% (50/12,386), with all detected variants at the S768 site being S768I. In contrast, the rate of *EGFR* S768 variants detected by NGS was 1.15% (96/8345). Compared with NGS, the detection rate of ARMS-PCR was significantly lower (0.40% vs. 1.15%, *p* < 0.001; RD = −0.007, 95% CI: −0.010–−0.005; RR = 0.351, 95% CI: 0.250–0.493). Beyond S768I, NGS identified additional variant types at the S768 site, including S768_D770dup, S768_V769insLDS, and S768_V769delinsIL, accounting for 33.33% (32/96), 1.04% (1/96), and 2.08% (2/96) of NGS-detected S768 variants, respectively. Due to the limitation of ARMS-PCR in distinguishing specific forms of *EGFR* ex20ins variants, insertions at the S768 site and other sites of exon 20 all appeared as elevated amplification curves for *EGFR* ex20ins variants. Therefore, we further focused on the rate of *EGFR* S768I missense mutation and found that the rate of S768I detected by ARMS-PCR was also significantly lower than that detected by NGS (61/8345) (0.40% vs. 0.73%, *p* = 0.002; RD = −0.003, 95% CI: −0.005–−0.001; RR = 0.552, 95% CI: 0.380–0.802).

### 3.3. Analysis of EGFR S768I Variants Co-Occurring with Other EGFR Mutations

**Figure 1:**
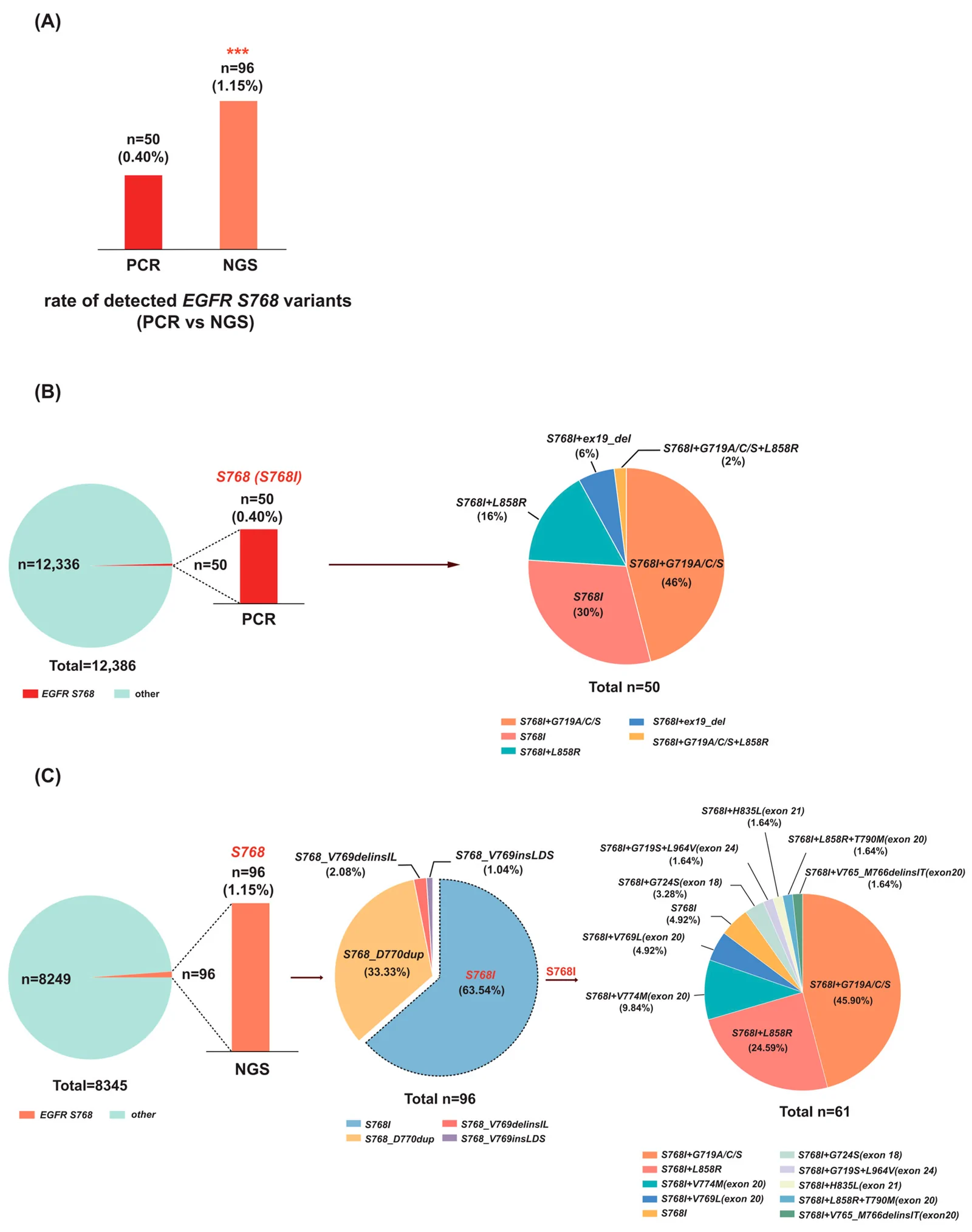
*EGFR* S768 and co-occurring mutations detected by amplification refractory mutation system polymerase chain reaction (ARMS-PCR) and Next-Generation Sequencing (NGS) in non-small cell lung cancer (NSCLC). (**A**) Rate difference of *EGFR* S768 detected by ARMS-PCR and NGS. The asterisk (***) indicates *p* < 0.001; (**B**) *EGFR* S768 and co-occurring mutations detected by ARMS-PCR; (**C**) *EGFR* S768 and co-occurring mutations detected by NGS.

In the primary specimen-level analysis, the rate of *EGFR* S768I variants co-occurring with other *EGFR* mutations detected by ARMS-PCR also differed from that detected by NGS. Among the 50 samples with ARMS-PCR detected S768I, 35 concurrently harbored other *EGFR* mutations, accounting for 70.00% (35/50). The concomitant *EGFR* mutations detected by ARMS-PCR alongside S768I were G719A/C/S, L858R, and ex19_del, accounting for 48.00% (24/50), 18.00% (9/50), and 6.00% (3/50), respectively. In contrast, among the 61 samples with NGS-detected S768I variants, 58 concurrently harbored other *EGFR* mutations (95.08%, 58/61). Considering the different coverage between two methods, we then restricted the analysis to variants within the shared coverage of both NGS and ARMS-PCR, NGS (45/48) still detected a significantly higher co-occurrence rate than that observed with ARMS-PCR (93.75% vs. 70.00%, *p* = 0.002; RD = 0.238, 95% CI: 0.093–0.382; RR = 1.339, 95% CI: 1.101–1.629). Additionally, NGS also identified S768I variants co-occurring with other *EGFR* mutations beyond G719A/C/S and L858R, which reflected its broader panel coverage (Figure 1). However, the co-occurrence rates of S768I with G719A/C/S and with L858R detected by NGS were 47.54% (29/61) and 26.22% (16/61), respectively, showing no significant difference compared to ARMS-PCR (*p* = 0.96, *p* = 0.30).

NGS could also detect concurrent *EGFR* S768I and *EGFR* copy number amplification, with a co-occurrence rate of 14.75% (9/61), significantly higher than that of ARMS-PCR, as ARMS-PCR failed to detect copy number amplification. Interestingly, insertion at the S768 site rarely coexisted with other *EGFR* mutations. Only one case of an insertion at the S768 site coexisted with other *EGFR* mutation (*EGFR* ex19_del), a rate significantly lower than that of S768I missense mutation co-occurring with other *EGFR* mutations (3.03% vs. 95.08%, *p* < 0.001; RD = −0.921, 95% CI: −1.000–−0.841; RR = 0.032, 95% CI: 0.005–0.220). However, due to the small number of insertion cases, these effect estimates should be interpreted with caution.

Additionally, to address the potential non-independence from repeated specimens, a patient-level sensitivity analysis restricted to the first specimen showing *EGFR* S768I variant per patient was performed. Only 2 and 0 repeated specimens were removed from NGS and ARMS-PCR group, respectively. The conclusion of patient-level analysis was consistent with the primary specimen level analysis (Supplementary Table S1). Given the sex imbalance observed in the S768I subgroup, sex-stratified analyses were conducted. NGS consistently detected more co-mutations than ARMS-PCR in both male (91.3% vs. 70.59%) and female (96% vs. 68.75%) subgroups, but these findings were exploratory and should be interpreted as descriptive observations due to the limited sample size (57 for male and 41 for female with *EGFR* S768I and co-occurred variants within the shared coverage). Detailed results were provided in Supplementary Tables S2 and S3. Overall, these findings showed consistent numerical trends, indicating that the observed difference in co-occurrence rates was unlikely to be driven solely by repeated testing or sex imbalance.

### 3.4. Cases with Discordant ARMS-PCR and NGS Results

**Figure 2:**
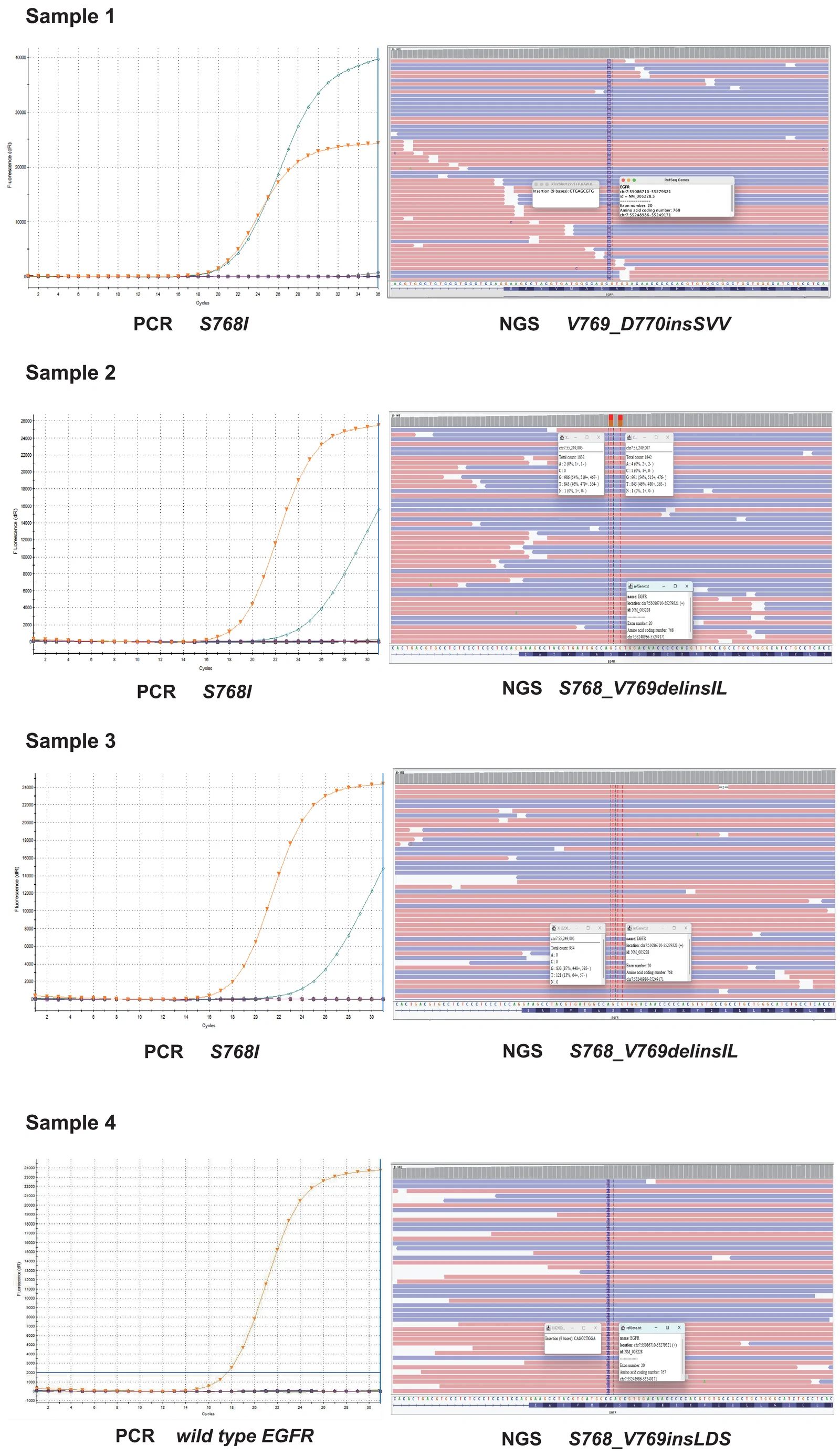
Inconsistence between amplification refractory mutation system polymerase chain reaction (ARMS-PCR) and Next-Generation Sequencing (NGS) in detecting *EGFR S768* special mutations of non-small cell lung cancer (NSCLC).

**Figure 3:**
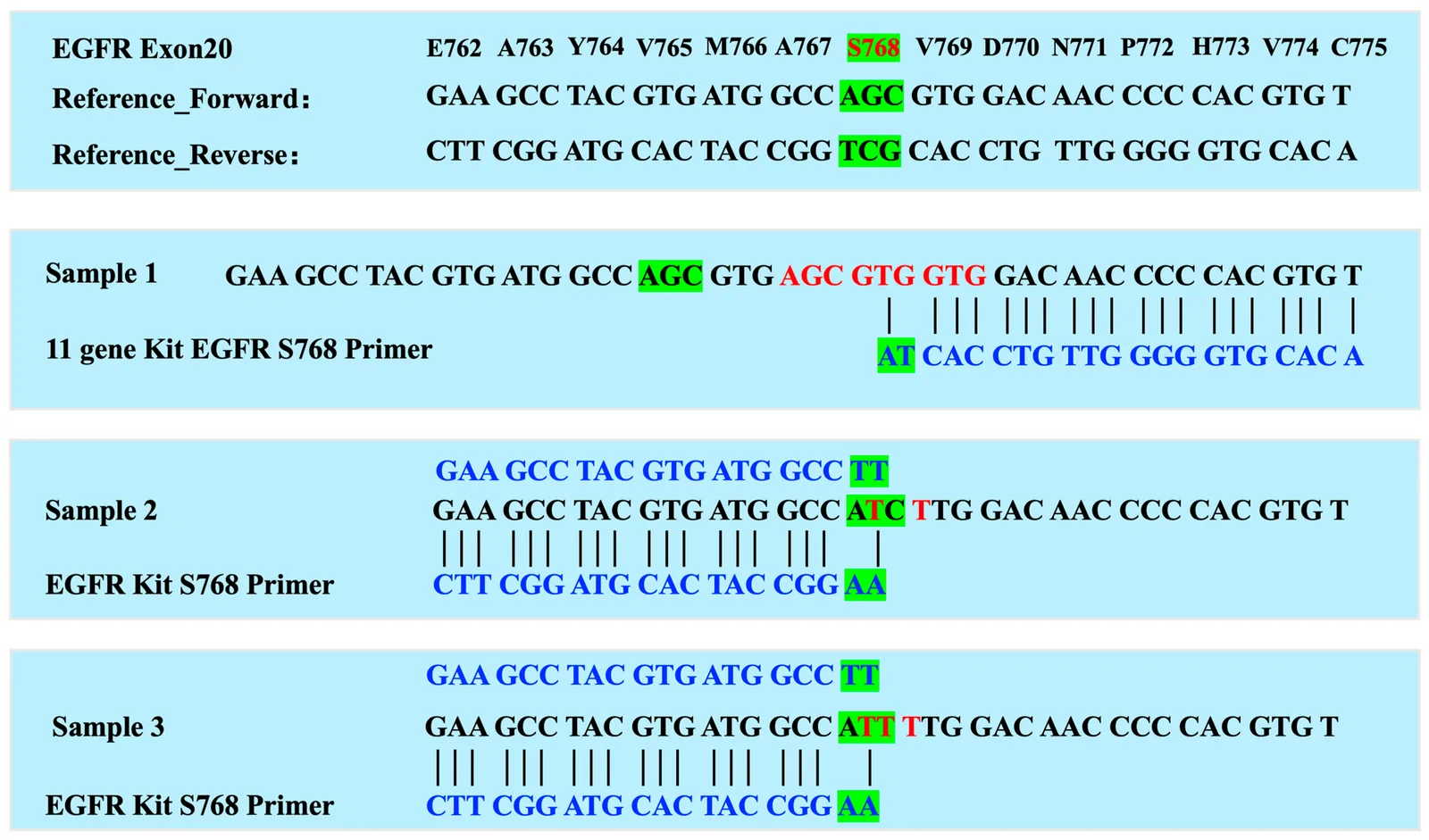
*EGFR* S768I primers of amplification refractory mutation system polymerase chain reaction (ARMS-PCR) bind to *EGFR* mutations in Patient-1, -2 and -3. Note: Red bases are mutations, blue bases are primers and letters with green background are S768 genetic code.

Among the 50 samples that tested positive for *EGFR* S768I by ARMS-PCR, three samples were identified by NGS as harboring V769_D770insSVV (c.2307_2308insAGCGTGGTG), S768_V769delinsIL (c.2303_2305delinsTCT), and S768_V769delinsIL (c.2303_2305delinsTTT), respectively. Another sample was detected negative for *EGFR* by ARMS-PCR but was identified as S768_V769insLDS (c.2304_2305insCTGGACAGC) by NGS, which was outside the coverage of the ARMS-PCR assay (Table 2, Figure 2). By carefully comparing the primer and mutation sequence, we found that only one mismatch between primer and each mutation, suggesting complex mutations or insertions around the *EGFR* S768 site cause discordant results when using ARMS-PCR possibly due to non-specific primer binding (Figure 3).

### 3.5. Clinicopathological Characteristics and Treatment of Cases with Discordant Results

Among these four patients with discordant ARMS-PCR and NGS results, the mean age was 53.8 ± 9.2 years, with three females and one male. All patients were diagnosed with lung adenocarcinoma. Three were classified as having advanced-stage (IV) disease and one as early-stage (IA3) (Table 3).

Patient-1 was diagnosed with pulmonary adenocarcinoma through biopsy of the primary pulmonary lesion and cervical lymph node metastases, with clinical stage cT3N3M1. The cervical lymph node metastatic lesion was submitted for molecular pathological testing by ARMS-PCR, which detected an *EGFR* S768I mutation. Therefore, icotinib combined with radiotherapy was selected as the initial treatment. Five months later, the patient developed disease progression with adrenal metastases. After switching to afatinib for 2.6 months, disease progression was again observed. The patient subsequently underwent a biopsy of the supraclavicular lymph node lesion, and the tissue specimen was submitted for NGS analysis. NGS did not detect *EGFR* S768I; instead, it identified an *EGFR* V769_D770insSVV variant. To explore the reason for the inconsistency, we tested the supraclavicular lymph node metastatic lesion by ARMS-PCR, and it still showed *EGFR* S768I. We then further tested the previous cervical lymph node metastatic lesion by NGS and confirmed it was *EGFR* V769_D770insSVV. Therefore, we considered that the possible reason for this discordance may be due to non-specific binding of the S768I primer of ARMS-PCR to the *EGFR* V769_D770insSVV (c.2307_2308insAGCGTGGTG) sequence during the PCR reaction (Figure 3). Based on the NGS result, the treatment regimen of Patient-1 was adjusted to pemetrexed plus carboplatin (PC) in combination with ivonescimab (PD-1/VEGF bispecific antibody). At the patient’s most recent follow-up, no disease progression had been observed for nearly six months since the treatment change.

Patient-2 and Patient-3 tested positive for *EGFR* S768I by ARMS-PCR, but no other concomitant *EGFR* mutations were identified. Given the high frequency of *EGFR* S768I co-occurring with other *EGFR* mutations, we performed retesting using NGS. Both cases were found to harbor *EGFR* S768_V769delinsIL. As no drugs targeting *EGFR* ex20ins variants were approved in 2022, Patient-2 continued with afatinib as treatment. After 3.3 months of afatinib monotherapy, due to poor efficacy and poor drug tolerance, the regimen was switched to afatinib plus Tegafur, Gimeracil and Oteracil Potassium Capsules. After two months, the regimen was changed to afatinib plus anlotinib, followed by anlotinib and almonertinib monotherapy. These frequent drug switches suggested poor efficacy of EGFR-TKIs, ultimately leading to one cycle of furmonertinib (third-generation EGFR-TKI) treatment, after which the patient was lost to follow-up. Patient-3 did not receive targeted therapy after surgical resection due to early-stage disease.

**Figure 4:**
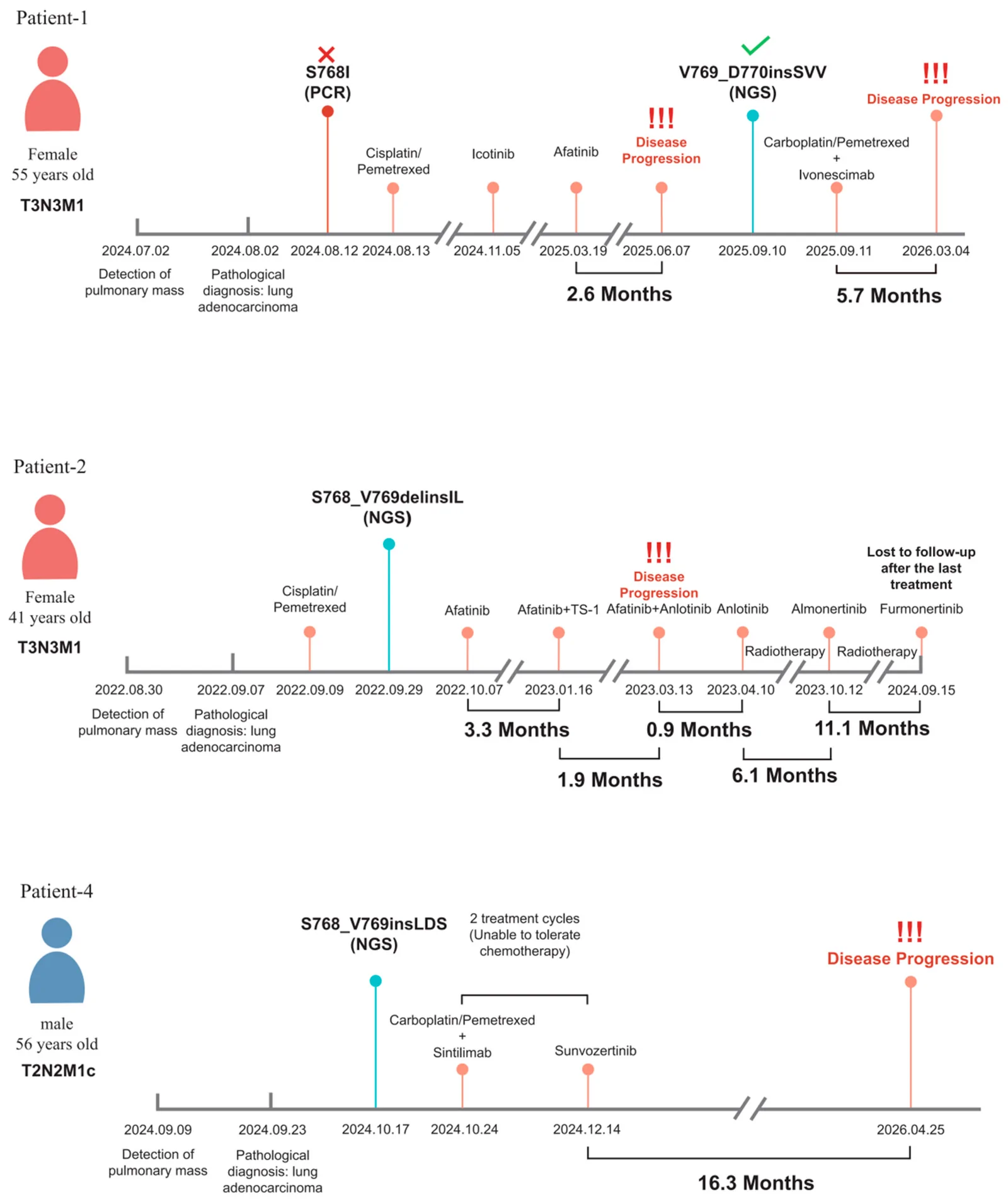
Treatment timeline for Patient-1, Patient-2, and Patient-4 with non-small cell lung cancer (NSCLC).

Patient-4 tested negative for *EGFR* mutations by ARMS-PCR but was identified as harboring *EGFR* S768_V769insLDS by NGS. Pemetrexed plus carboplatin combined with sintilimab (a PD-1 inhibitor) was initially selected as the treatment regimen. After two cycles of this therapy, the regimen was switched to sunvozertinib (targeting *EGFR* ex20ins variants) due to patient intolerance. As of the most recent follow-up, the patient has remained stable for 16.3 months (Figure 4).

## 4. Discussion

Different *EGFR* mutation types confer differential sensitivity to TKIs, making their accurate detection critical for personalized treatment in patients with NSCLC. Although rare, missense mutations and insertion mutations at the *EGFR* S768 site have entirely distinct treatment strategies, carrying important clinical implications. In this retrospective study of 20,731 NSCLC specimens, the rate of *EGFR* S768 variants detected by ARMS-PCR was significantly lower than that detected by NGS. Moreover, ARMS-PCR possibly causes discordant results with NGS when detecting complex mutations or insertions at or nearby the *EGFR* S768 site, which may influence treatment decisions and possibly affect patient prognosis.

ARMS-PCR has the advantage of short detection turnaround time and low cost, making it widely used in molecular pathological diagnosis. However, due to the limited number of sites it covers, ARMS-PCR could not detect all *EGFR* mutation types. The narrower coverage of ARMS-PCR may partly explain the lower detection rate for variants at the *EGFR* S768 site in NSCLC compared to NGS. *EGFR* ex20ins variants are typically mutually exclusive with classic *EGFR* mutations and rarely harbor concurrent point mutations at other *EGFR* sites [18]. In contrast, most *EGFR* S768I variants frequently co-occur with G719X or L858R mutations [5,19], consistent with our findings. Furthermore, the rate of S768I co-occurring with other *EGFR* mutations detected by ARMS-PCR was significantly lower than that detected by NGS. One reason was that NGS could identify S768I variant co-occurring with other *EGFR* mutations beyond the coverage of ARMS-PCR. The other possible reason is that some cases were detected as S768I by ARMS-PCR due to possible non-specific primer binding but were actually *EGFR* exon 20 insertions or complex mutations. Because these variants are typically not accompanied by concurrent *EGFR* mutations, this may lower the co-occurrence rate detected by ARMS-PCR. Given the high frequency of S768I variants co-occurring with other *EGFR* mutations, NGS may be recommended for validation when only *EGFR* S768I is detected by ARMS-PCR, especially for NSCLC patients (e.g., Patient-1 and Patient-2) who experience short-term disease progression after S768I-targeted TKI therapy such as afatinib, for whom NGS re-evaluation could reduce the risk of possible inaccurate S768I detection by ARMS-PCR.

Common *EGFR* ex20ins variants such as A767_V769dup and S768_D770dup can generally be identified by PCR. However, the proportion of patients with *EGFR* ex20ins variants detectable solely by PCR ranges from 11.8% to 58.9%, indicating a relatively high rate of missed detection [15]. In this study, one sample with no *EGFR* mutation detected by ARMS-PCR was identified as S768_V769insLDS by NGS. This is because the ARMS-PCR kits we used were designed to cover only the following mutations at the *EGFR* S768 site—S768I, S768_D770dup, and A767_S768insSLD—whereas S768_V769insLDS was not included. Furthermore, three samples in this study were detected as *EGFR* S768I variants by the ARMS-PCR platform, but subsequent NGS analysis revealed the variants as V769_D770insSVV and S768_V769delinsIL, indicating potential suboptimal performance of the ARMS-PCR platform. These findings demonstrated that ARMS-PCR may yield possible discordant detection with NGS for certain specific mutations at or near the *EGFR* S768 site. We further analyzed the sequences of the mutations at the S768 sites—V769_D770insSVV and S768_V769delinsIL—including insertion of GTGAGCGTG between the V769 and D770 sites, and base changes from AGCGTG to ATTTTG or ATCTTG at the S768 and V769 sites. In contrast, the missense mutation S768I typically involves a base alteration from AGC to ATC at the *S768* site. Comparative analysis revealed that both S768_V769delinsIL and *S768I* harbored G > T substitutions at the 2303 base site, suggesting potential non-specific primer binding at the 3′ end. The V769_D770insSVV variant also involved a primer sequence fragment that highly matched with that of *EGFR* S768I with only one mismatch, the remaining primer sequence demonstrates strong binding stability, possibly resulting in erroneous extension (Figure 3). This may be attributable to limitations of the ARMS-PCR platform, such as non-specific binding of primers to some complex mutations or insertions around the *EGFR* S768 site. However, this remains a hypothetical explanation based on primer and mutation sequence comparison and requires further experimental validation. Furthermore, the diversity of atypical *EGFR* mutations and their increasing incidence further highlight the necessity of NGS testing [6].

For patients with rare *EGFR* mutations, such as variants at or near the S768 site, accurate identification of the mutation during initial treatment is undoubtedly crucial. In our study, Patient-1 experienced disease progression shortly after inappropriate targeted therapy, possibly due to inaccurate ARMS-PCR detection. Patient-2, after initially detected as S768I by ARMS-PCR, underwent NGS re-examination, which revealed the *EGFR* variant—S768_V769delinsIL. This variant involves the substitution of one leucine (L) following the replacing isoleucine (I) at the S768 site. Unfortunately, because no targeted drugs for *EGFR* ex20ins were approved in 2022, the therapeutic response to afatinib remained suboptimal. In contrast, Patient-4 initially tested negative for *EGFR* mutations by ARMS-PCR but was later identified as having an *EGFR* S768 insertion variant (S768_V769insLDS) through NGS retesting, thereby avoiding the risks associated with an ARMS-PCR result limited by its designed coverage. This patient received standardized treatment with sunvozertinib, which targets *EGFR* ex20ins variants. As of the most recent follow-up, no disease progression had been observed for 16.3 months, indicating well-controlled disease.

In this study, we reported several base sequence alterations associated with discordant results between ARMS-PCR and NGS at the *EGFR* S768 site. We also analyzed, through clinical cases, the potential implications of discordant results at the *EGFR* S768 site on treatment decisions, suggesting that such discordance warrants clinical awareness.

However, this study has several limitations.

First, our analysis was retrospective and all specimens were obtained from a single center. Detailed clinicopathological information, such as staging, pathological type, treatment, and prognosis, were not uniformly available in the retrospective database. Baseline comparison was therefore limited to age and sex. In addition, mutation detection rates were calculated based on submitted specimens rather than unique patient, because some patients contributed multiple specimens due to multi-lesions in different sites of lung or repeat sampling and testing before and after treatment. As a result, overall detection rates should be interpreted with caution. But for the *EGFR* S768I-positive specimens, a patient-level detection rate analysis including only the first specimen showing S768I per patient was performed, and the conclusion was consistent with the primary specimen-level analysis (Supplementary Table S1).

Second, this was a real-world retrospective clinic study which was not designed as a paired comparison because paired testing is not part of routine clinical practice. Paired ARMS-PCR and NGS testing was only performed for the four discordant specimens. As a result, potential selection bias cannot be excluded. To investigate the potential bias from non-paired cohorts, we compared the baseline characteristics between the two groups, which showed adequate balance in age (SMD = 0.060) and sex (SMD = 0.089) (Table 1). In the *EGFR* S768I subgroup, the sex distribution was imbalanced (SMD = 0.458). So we performed sex-stratified analyses, which descriptively showed higher co-occurrence rates detected by NGS in both sexes, though statistical power was limited. Although the large number of samples in this study (12,386 ARMS-PCR vs. 8345 NGS) provided real-world observations of platform differences, it did not resolve the selection bias inherent in the non-paired design. Prospective paired study is required for validation. Given this non-paired design and different assay coverage, the observed detection rates should not be interpreted as a direct measure of relative sensitivity.

Third, this retrospective clinical study collected data over a very long period from 2011 to 2025. The ARMS-PCR kits, NGS panels, and bioinformatics pipelines changed over time. Although the primer, probe, bioinformatic parameter for calling *EGFR* S768 variants have not changed, we still cannot fully rule out potential affecting factors. So prospective multi-center paired studies are needed for validation.

Finally, the research primarily focused on variants at or near the *EGFR* S768 site; whether ARMS-PCR detection of mutations at other *EGFR* sites may produce similar inaccuracy requires further validation.

## 5. Conclusions

Both ARMS-PCR and NGS are excellent in detecting *EGFR* S768I but for complex mutations or insertions around the *EGFR* S768 site, ARMS-PCR may give inaccuracy results possibly caused by non-specific primer binding. For patients with only the *EGFR* S768I detected by ARMS-PCR without any other *EGFR* mutations and experienced rapid disease progression during treatment with *EGFR* S768I-directed targeted therapy such as afatinib, NGS may be considered to confirm the mutation for guiding appropriate targeted therapy.

## Figures and Tables

**Table 1:** Baseline characteristics of ARMS-PCR and NGS groups.

	ARMS-PCR	NGS	SMD
*n*	12,386	8345	
Age, mean ± SD	60.30 ± 10.38	60.93 ± 10.44	0.060
Sex, *n* (%)			0.089
female	5354 (43.23%)	3978 (47.67%)	
male	7032 (56.77%)	4367 (52.33%)	

**Table 2:** Four cases with special *EGFR* S768 mutations.

Sample	Sample Type	Sample Source	ARMS-PCR Result	NGS Result	Other *EGFR* Mutations
1	Biopsy specimen	Metastatic lesion	*S768I*	*V769_D770insSVV* (c.2307_2308insAGCGTGGTG)	None
2	Biopsy specimen	Primary lesion	*S768I*	*S768_V769delinsIL* (c.2303_2305delinsTCT)	None
3	Surgical specimen	Primary lesion	*S768I*	*S768_V769delinsIL* (c.2303_2305delinsTTT)	None
4	Biopsy specimen	Primary lesion	Wild type *EGFR*	*S768_V769insLDS* (c.2304_2305insCTGGACAGC)	None

**Table 3:** Clinical and pathological characteristics of patients with discordant results.

Sample	Patient	Age	Gender	Tumor Type	Stage *
1	Patient-1	55	Female	lung adenocarcinoma	cT3N3M1 (IV)
2	Patient-2	41	Female	lung adenocarcinoma	cT3N3M1 (IV)
3	Patient-3	63	Female	lung adenocarcinoma	cT1N0M0 (IA3)
4	Patient-4	56	male	lung adenocarcinoma	cT2N2M1c (IVB)

* Staging was based on the AJCC 8th edition clinical TNM (cTNM) staging system for lung cancer.

## Data Availability

The main data has been included in the paper. Please contact Dr. Bo Huang (huangbo1131@126.com) or Prof. Xiu Nie (niexiuyishi@126.com) for additional information.

## Supplementary Materials

The following supporting information can be downloaded at: https://www.mdpi.com/article/10.3390/diagnostics16213525/s1, Table S1: Analysis of *EGFR* S768I co-occurrence rates in the S768I-positive subgroup within shared coverage of both ARMS-PCR and NGS (restricted to the first specimen per patient); Table S2: Baseline characteristics of ARMS-PCR and NGS groups with *EGFR* S768I variants; Table S3: Sex-stratified analysis of *EGFR* S768I co-occurrence rates.
