# Supplementary material for: Comprehensive Analysis of Next-Generation Sequencing and ARMS-PCR for Detecting EGFR S768 Mutations in Chinese Non-Small Cell Lung Cancer Patients
Source: Diagnostics (Basel). 2026 Oct 27;16(21):3525. doi: 10.3390/diagnostics16213525 (PMC13762321; doi:10.3390/diagnostics16213525)
Supplement: Supplementary file 1 [file diagnostics-16-03525-s001.zip › diagnostics-4571685-supplementary.pdf]

Supplementary Table S1. Analysis of *EGFR S768I* co-occurrence rates in the *S768I*-positive subgroup within shared coverage of both ARMS-PCR and NGS (restricted to the first specimen per patient).

Analysis	Method	N	Co-occurrence, n(%)	*p*	RD (95% CI)	RR (95% CI)
**specimen-level**	**ARMS-PCR**	**50**	**35 (70.00%)**	**0.002**	**-0.238 (-0.382, -0.093)**	**0.747 (0.614, 0.908)**
	**NGS**	**48**	**45 (93.75%)**			
**patient-level**	**ARMS-PCR**	**50**	**35 (70.00%)**	**0.003**	**-0.235 (-0.380, -0.089)**	**0.749 (0.615, 0.912)**
	**NGS**	**46**	**43(93.48%)**			

Supplementary Table S2. Baseline characteristics of ARMS-PCR and NGS groups with *EGFR S768I* variants.

	ARMS-PCR	NGS	*p*	SMD
**n**	**50**	**61**		
**Age, mean ± SD**	**61.18 ± 8.28**	**63.15 ± 9.20**	**0.244**	**0.225**
**Sex, n (%)**			**0.032**	**0.458**
**male**	**34 (68.00%)**	**28 (45.90%)**		
**female**	**16 (32.00%)**	**33 (54.10%)**		

Supplementary Table S3. Sex-stratified analysis of *EGFR S768I* co-occurrence rates.

Sex	Method	N	Co-occurrence, n(%)	*p*	RD (95% CI)	RR (95% CI)
**male**	**ARMS-PCR**	**34**	**24 (70.59%)**	**0.097**	**-0.207 (-0.399, -0.016)**	**0.773 (0.602, 0.994)**
	**NGS**	**23**	**21 (91.30%)**			
**female**	**ARMS-PCR**	**16**	**11 (68.75%)**	**0.026**	**-0.272 (-0.512, -0.033)**	**0.716 (0.510, 1.006)**
	**NGS**	**25**	**24 (96.00%)**			

*This sex-stratified analysis was performed at the specimen level to explore potential effect modification by sex. In total, 57 males and 41 females contain EGFR S768I and co-occurred variants within the shared coverage of ARMS-PCR and NGS. To address potential non-independence from repeated specimens, a patient-level detection rate analysis for the primary *S768I* co-occurrence comparison was separately performed (Supplementary Table S1), which showed consistent results.
